# DVIA technique for acute periprosthetic joint infection: a surgical technique and case series

**DOI:** 10.1186/s42836-026-00430-6

**Published:** 2026-09-08

**Authors:** Mingzhang Li, Boyong Wang, Zifei Yin, Changming Wang, Deyi Ding, Xiaohua Chen, Hao Shen, Qiaojie Wang

**Affiliations:** 1 Department of Orthopaedics, Shanghai Sixth People’s Hospital Affiliated to Shanghai Jiao Tong University School of Medicine, Shanghai, 200233 China; 2 Department of Orthopaedics, Kunshan Affiliated Hospital of Nanjing University of Chinese Medicine, Kunshan, 215300 Jiangsu China; 3 Department of Infectious Diseases, Shanghai Sixth People’s Hospital Affiliated to Shanghai Jiao Tong University School of Medicine, Shanghai, 200233 China

**Keywords:** Periprosthetic joint infection, Vacuum sealing drainage, DAIR, Vancomycin, Biofilm

## Abstract

**Aims:**

Periprosthetic joint infection (PJI) remains a major challenge in orthopaedic surgery. While debridement, antibiotics, and implant retention (DAIR) are a common treatment for acute PJI, its success rate is variable. Intra-articular antibiotic administration and wound healing techniques may enhance infection control and improve clinical outcomes. This study aimed to describe a modified technique combining DAIR with vacuum sealing drainage (VSD) and intra-articular antibiotic infusion (DVIA) for treating acute PJI, and evaluate its clinical feasibility and preliminary outcomes.

**Methods:**

This retrospective, single-center case series included 31 patients who underwent DVIA (hip: 19; knee: 12) between September 2019 and December 2023. Following DAIR, a technique integrating vacuum-sealed drainage for wound management with intra-articular antibiotic infusion was applied. Inflammatory markers (CRP, ESR) and intra-articular antibiotic concentrations were monitored at multiple time points. Treatment outcomes, CRP and ESR levels, Knee Society Score (KSS), KSS functional score, Harris Hip Score (HHS), and the 36-item Short-Form (SF-36) physical function scale were evaluated over a 12-month follow-up.

**Results:**

Infection control was achieved in 90.32% (28/31) of patients. CRP and ESR levels decreased after treatment. Joint function and patient-reported physical function improved during follow-up. Intra-articular vancomycin concentrations measured in a subset of patients exceeded previously reported minimum biofilm eradication concentration (MBEC) thresholds. No catheter-related complications, retrograde infections, or systemic antibiotic-related adverse events were observed.

**Conclusion:**

The DVIA technique appears to be a feasible adjunctive strategy for the management of acute PJI, enabling effective local antibiotic delivery and favorable clinical outcomes. These findings are preliminary and warrant further validation in prospective controlled studies.

## Introduction

Periprosthetic joint infection (PJI) following total hip arthroplasty (THA) or total knee arthroplasty (TKA) is a devastating, difficult-to-treat, and recurrent complication [1]. Current treatment options include debridement, antibiotics, and implant retention (DAIR), single-stage revision, two-stage revision, arthrodesis, and amputation [2, 3]. Among these, DAIR is a less invasive and cost-effective option, particularly for acute PJI, as it involves debridement and the replacement of modular components while preserving the remaining components of the prosthesis. However, its reported success rates vary widely, ranging from 30 to 90% [4–12], indicating inconsistent clinical efficacy.

Several factors may contribute to DAIR failure [13]. Among these, biofilm formation on prosthetic surfaces represents a critical mechanism underlying infection persistence [14]. Postoperative intravenous antibiotic therapy alone may result in drug concentrations at the infection site failing to reach the minimum biofilm eradication concentration (MBEC), potentially leading to pathogen dissemination, recolonization, and reinfection [15, 16]. Strategies enabling sustained intra-articular antibiotic delivery have been explored to improve local drug exposure. Mu et al. reported intra-articular antibiotic application for early postoperative infections; however, their study was limited to infections within 3 months after primary arthroplasty, with short drug exposure duration and without monitoring intra-articular antibiotic concentrations or functional outcomes [17]. Moreover, patients with acute PJI often present with skin defects and accumulation of exudates and necrotic tissue in the joint, which limit the effectiveness of intra-articular antibiotic therapy, delay wound healing, and impair joint function. Therefore, alongside intra-articular antibiotic therapy, adequate drainage and wound management are required as complementary measures to reduce microbial burden, maintain a stable local environment, and promote wound healing.

Based on these considerations, a modified treatment protocol for acute PJI was developed: vacuum sealing drainage (VSD)-assisted intra-articular antibiotic infusion combined with standard DAIR and systemic antibiotic therapy (DVIA). This study aims to describe the DVIA technique in detail and to report its preliminary clinical outcomes in patients with acute PJI, including infection control, inflammatory markers (CRP and ESR), intra-articular antibiotic concentrations, joint function (Knee Society Score [KSS], Harris Hip Score [HHS]), and patient-reported physical function (the 36-item Short-Form [SF-36]).

## Methods

### Study design

Following approval from the institutional review board, this retrospective case series included patients who were treated with the DVIA technique at a tertiary medical center between September 2019 and December 2023. The inclusion criteria were as follows: patients diagnosed with PJI following hip or knee arthroplasty, based on the Musculoskeletal Infection Society (MSIS) guidelines [18]. With reference to clinical classifications of early postoperative infections (Tsukayama type 2) and late hematogenous infections (Tsukayama type 3), patients with acute PJI suitable for DAIR were included. Specifically, this comprised acute infections occurring within 3 months after the index arthroplasty, or late hematogenous infections with symptom onset within 3 months in a previously well-functioning joint, regardless of the time since the index arthroplasty [19, 20]. Included patients were required to undergo at least 12 months of clinical follow-up after DAIR treatment, with complete medical records and follow-up data. The exclusion criteria were as follows: patients who did not meet the above diagnostic criteria, patients treated with one- or two-stage revision surgeries, and patients who had incomplete follow-up records.

### Surgical technique and pathogen identification

Synovial fluid was collected by preoperative joint aspiration for pathogen culture. A medial parapatellar approach was used for the knee, and a posterolateral approach for the hip. In cases where synovial fluid was not obtained preoperatively, joint aspiration was repeated during surgery to collect the synovial fluid for pathogen culture.

A thorough debridement was performed. The scar tissue, along with necrotic or infected bone and soft tissue, was excised. Peri-prosthetic tissue samples were collected for histopathological examination and microbial culture. Modular components, such as the knee’s polyethylene inserts, femoral head, and hip acetabular liner, were removed and placed in saline for ultrasonic oscillation (300W, 10 min), followed by pathogen culture of the ultrasonic fluid [21]. Component surfaces were mechanically scrubbed to disrupt biofilms. The surgical field was irrigated repeatedly with saline, 3% hydrogen peroxide, and 0.25% povidone-iodine. Iodine tincture-soaked gauze was then placed in the joint for 15 min. The surgeon changed gowns and gloves, replaced surgical instruments, and redraped the patient. The gauze was removed from the wound, and the joint was irrigated again with saline, 3% hydrogen peroxide, and iodine tincture. After confirming the removal of any residual infected or necrotic tissue, new modular components were implanted, and the wound was sutured.

**Fig. 1:**
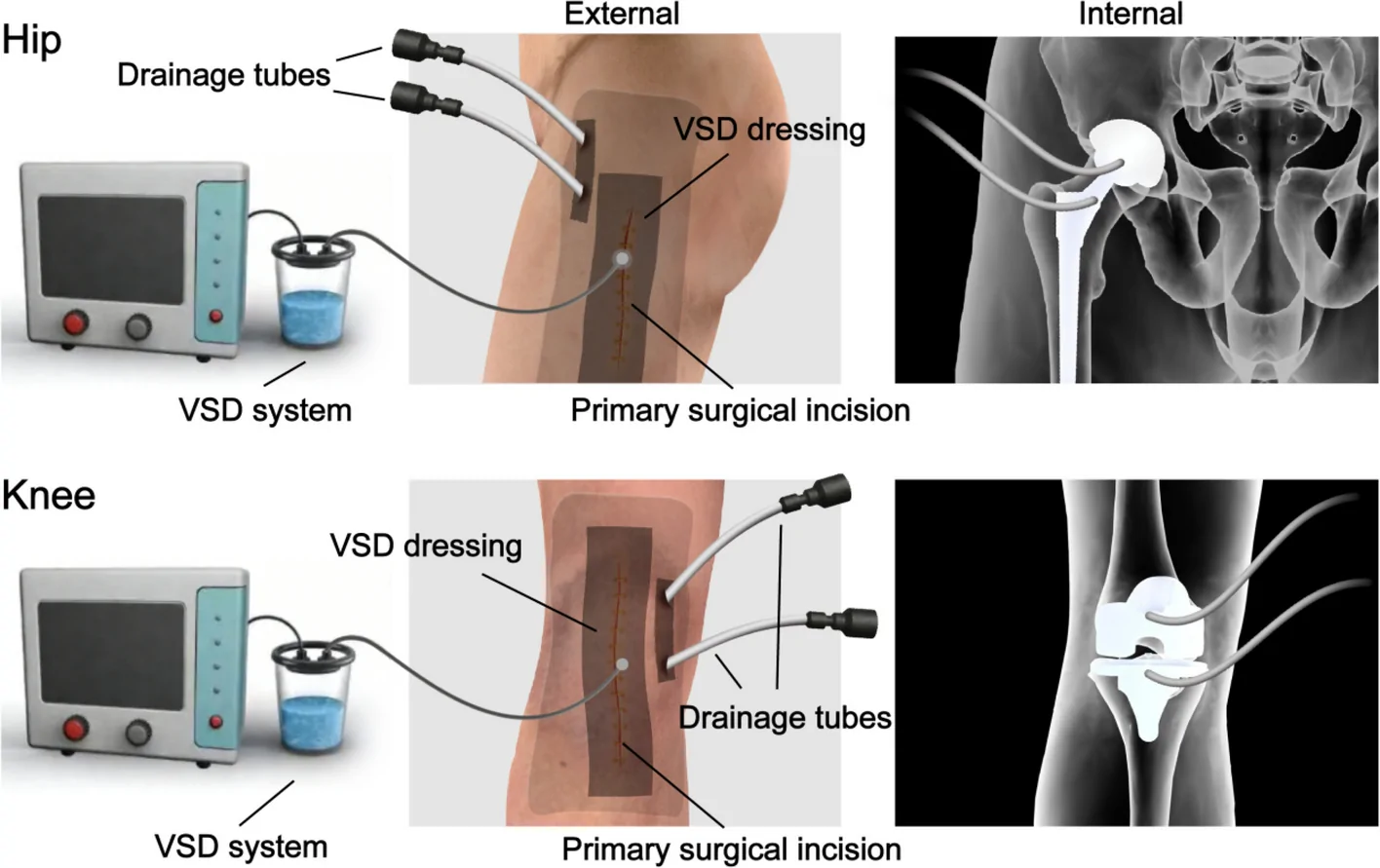
Schematic diagram of DVIA treatment

For DVIA, after standard DAIR debridement and implantation of new modular components, two medical-grade silicone drainage tubes were placed into the joint cavity through separate small stab incisions adjacent to the primary surgical incision, rather than directly through the incision. These two tubes functioned as intra-articular drainage and infusion channels and were independent of the VSD negative-pressure circuit. The incision and superficial wound area were then covered with a polyurethane VSD dressing (VAC®, Wuhan VSD Medical Science & Technology Co., Ltd., China) without any structural modification, and continuous negative pressure (150 mmHg) was applied only to the superficial wound space, without direct communication with the intra-articular cavity. The joint tubes passed through the polyurethane VSD dressing foam and were sealed by the adhesive transparent drape, providing a sealed and sterile environment while maintaining separation from the VSD suction system (Fig. 1).

All procedures were performed by a dedicated orthopaedic team following standardized protocols. All microbial cultures were performed according to standard laboratory protocols, including preoperative and intraoperative synovial fluid, intraoperative tissue samples, and ultrasonic fluid from modular components. Cases in which no pathogen was isolated from the above samples were classified as culture-negative PJI. Treatment regimens for culture-negative cases followed the same protocol as culture-positive cases to ensure consistency in clinical management.

### Antibiotic application

For patients with identified pathogens, systemic antibiotic therapy was selected according to pathogen identification, antimicrobial susceptibility testing, and clinical judgement. Levofloxacin was used empirically when the pathogen was unidentified. Systemic antibiotic therapy typically consisted of intravenous administration for 2 weeks, followed by oral therapy for 6 weeks.

In addition to systemic antibiotic therapy, intra-articular antibiotic infusion was performed once daily through the drainage tubes. For intra-articular administration, vancomycin was used for monomicrobial Gram-positive bacterial infections, whereas meropenem was used for monomicrobial Gram-negative bacterial infections. In cases requiring broader antimicrobial coverage, such as polymicrobial infections, fungal infections, or pathogen-unidentified cases, combination intra-articular therapy with vancomycin plus meropenem was applied.

Before each infusion, the connector site was disinfected and accumulated joint effusion was aspirated. Subsequently, 1 g of vancomycin or meropenem dissolved in 20 mL of normal saline was infused into the joint cavity as a single daily dose. For patients with baseline creatinine clearance (CrCl) < 50 mL/min, the intra-articular antibiotic dose was reduced as follows: 750 mg for CrCl 30–49 mL/min, and 500 mg for CrCl < 30 mL/min. After infusion, the intra-articular drainage tubes were clamped for approximately 24 h to allow antibiotic retention within the joint cavity. The VSD system continued negative-pressure drainage of the superficial wound and remained separate from the intra-articular drainage system. Before the next daily infusion, the drainage tubes were reopened, accumulated intra-articular fluid was aspirated, and the subsequent antibiotic dose was administered.

This cycle was continued for 12–14 days, corresponding to the typical indwelling duration of the drainage tubes in clinical practice [22, 23]. Intra-articular antibiotic infusion was discontinued upon drainage tube removal when clinical signs of infection had improved, wound conditions were acceptable, and no further surgical intervention was required. Antibiotic-related adverse events were managed according to clinical evaluation, including adjustment or discontinuation of the suspected offending agent when necessary.

### Demographics

**Fig. 2:**
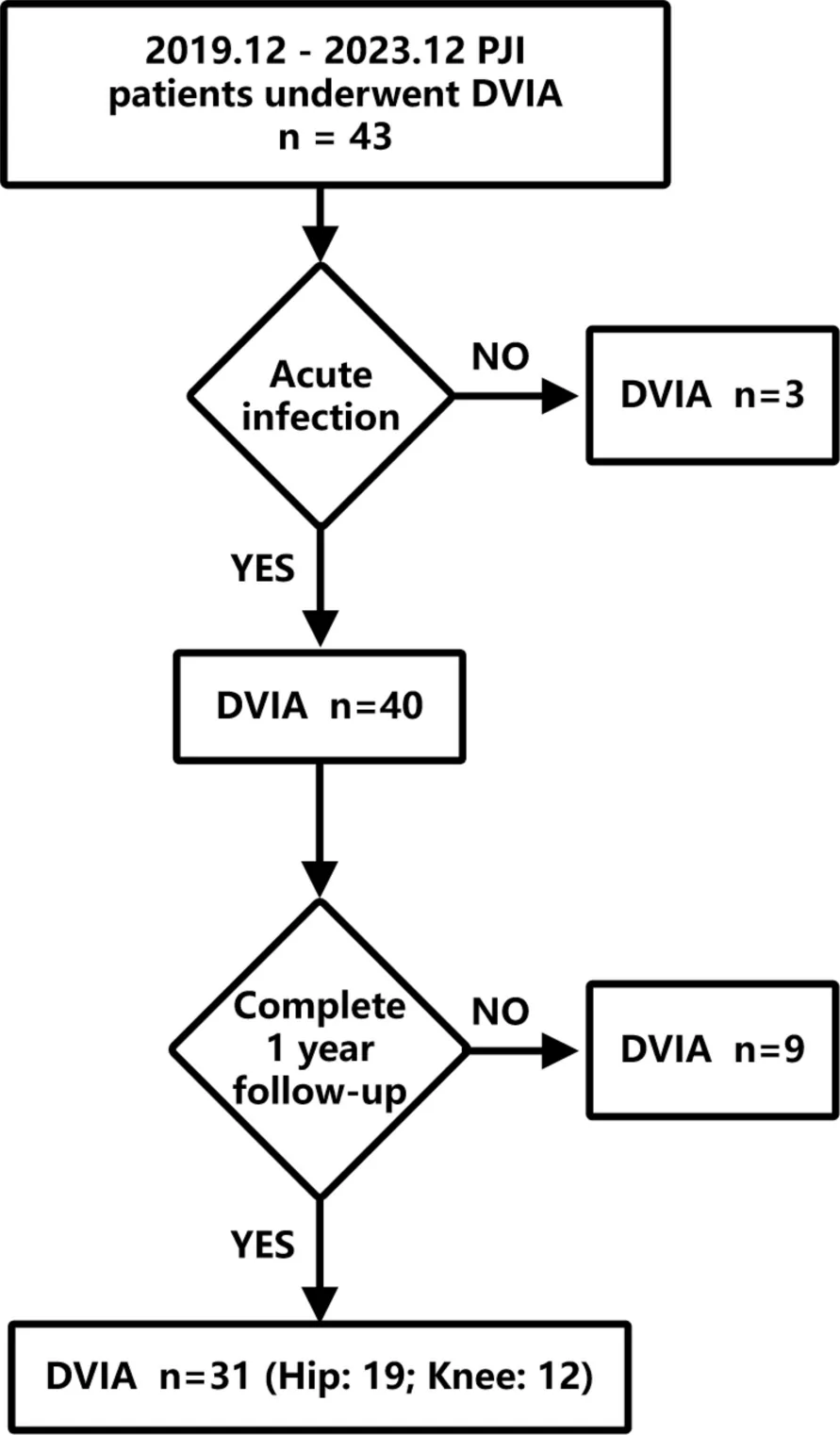
Flowchart of patient selection

A total of 43 patients underwent DVIA treatment at our center between September 2019 and December 2023. After applying the inclusion and exclusion criteria described above, 31 patients were included in the final analysis (hip: 19; knee: 12) (Fig. 2). Patient demographics and clinical characteristics, including age, gender, BMI, affected side, infected joint, underlying diseases, age-adjusted Charlson Comorbidity Index (aCCI), infection type, pathogens, and antibiotic regimens, were recorded and summarized in Table 1. The specific pathogens and corresponding postoperative antibiotic treatment regimens are listed in Table 2.

**Table 1:** Demographics Characteristics

Variables	DVIA *N* = 31
Age (y), M (P25, P75)	65 (58.00, 72.00)
BMI, Mean (SEM)	25.49 (0.68)
Sex, *n* (%)	
Women	12 (38.71)
Men	19 (61.29)
Laterality, *n* (%)	
Left	13 (41.94)
Right	17 (54.84)
Bilateral	1 (3.22)
Joint, *n* (%)	
Knee	12 (38.71)
Hip	19 (61.29)
Underlying diseases, *n* (%)	
Hypertension	14 (45.16)
Diabetes mellitus	5 (16.13)
Hepatic or renal diseases	9 (29.03)
Immune system diseases	3 (9.68)
aCCI, Mean (SEM)	3.52 (0.31)
Time to infection after arthroplasty (m), M (P25, P75)	2.00 (0.25, 8.00)
Acute infection type, *n* (%)	
Early postoperative infection	19 (61.29)
Late hematogenous infection	12 (38.71)
Clinical manifestation, *n* (%)	
Rest pain	24 (77.42)
Sinus tract and pus	15 (48.39)
Elevated skin temperature	22 (70.97)
Limited range of motion	22 (70.97)

M, median; P25, 25th percentile; P75, 75th percentile; SEM, standard error of the mean; BMI, body mass index; aCCI, age-adjusted Charlson Comorbidity Index

Note: Time to infection after arthroplasty was defined as the interval from the index arthroplasty to the onset of infection symptoms

**Table 2:** Pathogens, antibiotic treatment regimens, and outcome

Number	Pathogen	Irrigation	Intravenous	Oral	Outcome
1	CoNS	V	L	L + Rifampicin	Success
2	S. aureus	V	V	L + Rifampicin	Success
3	S. aureus	V	Linezolid	L + Rifampicin	Success
4	S. aureus + Acinetobacter sp. + Moraxella sp.	V + M	L	L + Rifampicin	Success
5	CoNS + Acinetobacter sp. + Fungi	V + M	Cefoperazone Sodium/Sulbactam Sodium + Fluconazole	L + Fluconazole	Success
6	Streptococcus sp.	V	L	L	Success
7	S. aureus	V	V	L + Rifampicin	Success
8	CoNS	V	L	L + Rifampicin	Success
9	CoNS	V	L	L + Rifampicin	Success
10	S. aureus	V	V	L + Rifampicin	Success
11	Unidentified	V + M	L	L	Success
12	Citrobacter sp. + Paenibacillus sp. + Fungi	V + M	L + Fluconazole	Amoxicillin + Fluconazole	Success
13	S. aureus	V	Moxifloxacin	Moxifloxacin	Success
14	P. aeruginosa + Cutibacterium sp. + Acinetobacter sp.	V + M	L	/	Success
15	S. aureus	V	L	L + Rifampicin	Success
16	Unidentified	V + M	L	L	Success
17	Fungi	V + M	Caspofungin	Caspofungin	Success
18	Streptococcus sp.	V	Moxifloxacin	Moxifloxacin	Success
19	Unidentified	V + M	L	L	Success
20	Klebsiella sp. + Ewingella sp.	V + M	Fosfomycin sodium	Faropenem	Success
21	P. aeruginosa + Serratia sp. + Fungi	V + M	Cefoperazone + Fluconazole	L + Fluconazole	Success
22	S. aureus + Enterobacter sp.	V + M	L	L + Rifampicin	Success
23	Streptococcus sp.	V	V	Linezolid	Success
24	S. aureus	V	V	Linezolid	Success
25	S. aureus	V	L	L	Success
26	S. aureus	V	L	L + Rifampicin	Success
27	S. aureus	V	V	L + Rifampicin	Success
28	Acinetobacter sp. + Klebsiella sp.	V + M	Ceftazidime	Minocycline	Success
29	S. aureus	V	L	Contezolid	Failure
30	Streptococcus sp.	V	Ceftriaxone sodium	Amoxicillin	Failure
31	S. aureus + Klebsiella sp.	V + M	L + Piperacillin sodium	/	Failure

CoNS, coagulase-negative staphylococci; S. aureus, Staphylococcus aureus; P. aeruginosa, Pseudomonas aeruginosa; sp., species; V, vancomycin; M, meropenem; L, levofloxacin

### Efficacy evaluation

Clinical outcomes were evaluated as a descriptive case series. Infection-control survival was recorded. The time to recurrence was defined as the interval between the index surgery and the first documented recurrence of infection or the end of the 1-year follow-up. Patients who did not experience recurrence during follow-up were censored at the last visit. CRP and ESR were measured preoperatively and after antibiotic therapy, with longitudinal trends monitored during the 12–14 days of DVIA treatment. Additionally, residual joint fluid was collected from the drainage tube using a syringe immediately before infusion at a consistent time on each sampling day to monitor intra-articular vancomycin concentrations, which were analyzed in the hospital laboratory. These measurements were performed as an exploratory pharmacokinetic assessment in a subset of patients. The KSS, KSS functional score, HHS, and the physical function scale score (PF) of SF-36 were assessed preoperatively, at 3 months postoperatively, and at the final follow-up visit. Treatment failure was defined as follows: the recurrence of infection symptoms, such as swelling, exudate, purulence, new sinus tracts, or rest pain during follow-up; the need for additional surgery due to infection (e.g., extra need for DAIR, debridement, or one- or two-stage revisions); prolonged antibiotic suppression therapy; recurrence of infection with the same pathogen; reinfection with different microorganisms; or infection-related mortality [24]. Treatment success was defined as the presence of normal inflammatory markers and the absence of clinical or imaging evidence of local inflammation, without the need for suppressive antibiotics or further surgery, during the 1-year follow-up.

### Data analyses

As this study is a descriptive case series, statistical analysis was primarily used for data summarization. Continuous variables are presented as mean ± standard error of the mean (SEM) or medians (25th and 75th percentiles) depending on distribution, and categorical variables are reported as counts and percentages. Longitudinal trends of laboratory markers and joint function scores are described graphically.

## Results

### Efficacy assessment

**Fig. 3:**
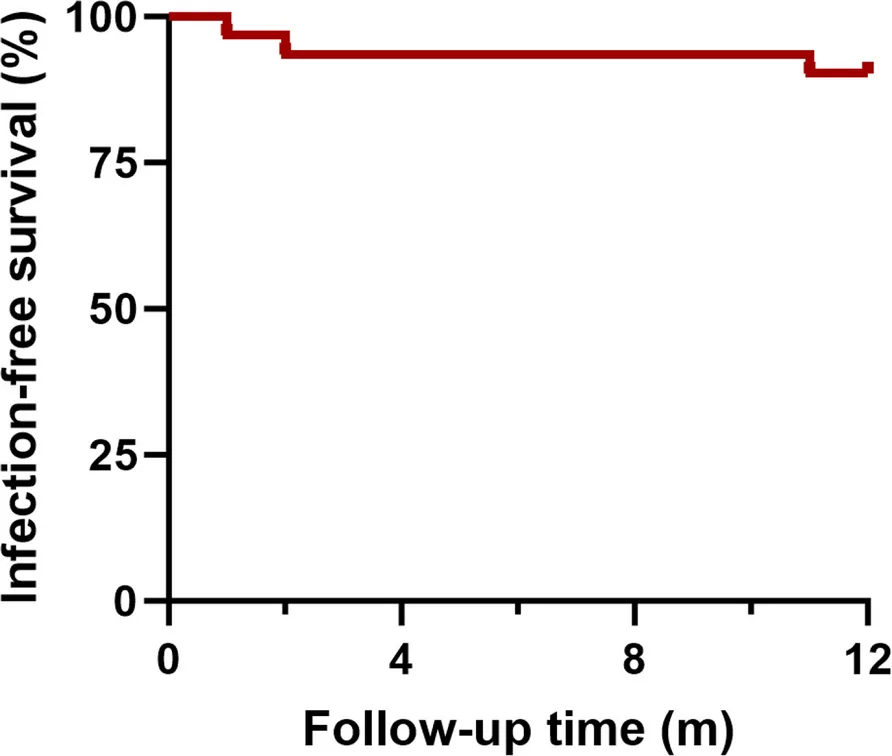
Kaplan–Meier analysis of infection-control survival within 12 months after DVIA treatment (*n* = 31; three infection-control failure events)

A total of 31 patients underwent DVIA treatment and were followed for 12 months. Three patients experienced infection-control failure during follow-up (9.68%), including one case requiring knee amputation due to uncontrolled infection (Table 3). Kaplan–Meier analysis estimated a 12-month infection-control rate of 90.32%, corresponding to an overall success proportion of 28/31 patients **(**Fig. 3). No patients experienced persistent exudates around the drainage tube or retrograde infections caused by the drainage tube. In the subset of patients with serial measurements, CRP and ESR demonstrated a decreasing trend during therapy. Joint function scores showed increasing trends over follow-up. KSS, KSS functional score, HHS, and the SF-36 PF scale all increased from baseline to 3 months postoperatively and to the final follow-up, suggesting improvement in joint mobility and daily physical function.

**Table 3:** Assessment of clinical efficacy

Variables	DVIA *N* = 31
Infection-control rate, n (%)	28 (90.32)
Inflammatory indicators	
Before surgery	
CRP (mg/L), M (P25, P75)	75.70 (25.60, 131.90)
ESR (mm/h), Mean (SEM)	76.51 (5.36)
After antibiotic treatment	
CRP (mg/L), M (P25, P75)	7.94 (2.11, 14.20)
ESR (mm/h), M (P25, P75)	30.50 (17.00, 69.25)
KSS	*n* = 12
Before surgery, Mean (SEM)	28.58 (1.49)
3 months after surgery, Mean (SEM)	70.36 (2.20)
Last follow-up, M (P25, P75)	82.00 (79.00, 86.00)
KSS Functional Score	*n* = 12
Before surgery, Mean (SEM)	25.42 (4.06)
3 months after surgery, Mean (SEM)	48.64 (2.87)
Last follow-up, Mean (SEM)	72.73 (2.64)
HHS	*n* = 19
Before surgery, M (P25, P75)	34.28 (26.45, 41.25)
3 months after surgery, Mean (SEM)	75.79 (1.07)
Last follow-up, Mean (SEM)	80.08 (0.86)
SF-36 PF	*n* = 31
Before surgery, M (P25, P75)	25.00 (20.00, 30.00)
3 months after surgery, M (P25, P75)	50.00 (45.00, 55.00)
Last follow-up, M (P25, P75)	55.00 (50.00, 80.00)

M, median; P25, 25th percentile; P75, 75th percentile; SEM, standard error of the mean; CRP, C-reactive protein; ESR, erythrocyte sedimentation rate; KSS, Knee Society Score; HHS, Harris Hip Score; SF-36, the MOS 36-Item Short-Form Health Survey; PF, physical functioning

### Changes in indicators after treatment

**Fig. 4:**
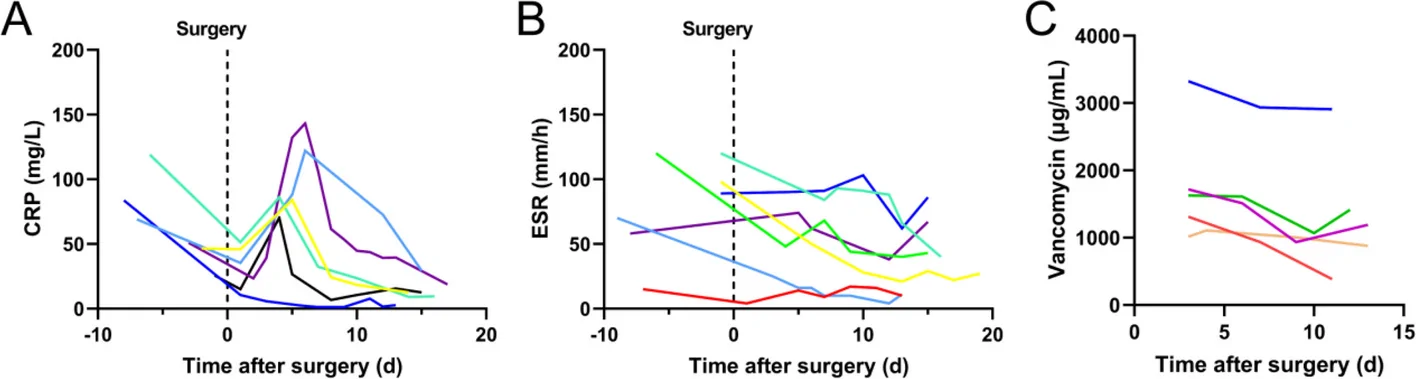
Changes in Inflammatory markers and intra-articular vancomycin concentrations in DVIA patients. **A** CRP changes at different time points in patients receiving DVIA treatment (*n* = 6). **B** ESR changes at different time points in patients receiving DVIA treatment (*n* = 7). **C** Changes in intra-articular vancomycin concentrations over time in DVIA patients (*n* = 5)

The levels of CRP, ESR, and intra-articular vancomycin concentrations were measured at different time points in several patients receiving DVIA treatment, and line charts depicting these changes over time were generated (Fig. 4). Preoperatively, CRP levels were elevated in all infected patients. Two days post-surgery, the CRP levels decreased compared with the preoperative measurements. Between days 3 and 6 postoperatively, CRP levels increased again in most patients, followed by a rapid decline, while one patient showed a continuous decrease in CRP levels without any subsequent increase. ESR monitoring showed that preoperative ESR levels were elevated. Although some patients experienced a slight rebound in ESR between days 3 and 6 postoperatively, the changes were less pronounced than those observed in CRP levels. Overall, following DVIA treatment, CRP and ESR levels showed a downward trend during the monitoring period. In the exploratory pharmacokinetic assessment, intra-articular vancomycin concentrations measured in five patients exceeded previously reported MBEC thresholds for S. aureus (512 μg/mL) and S. epidermidis (256 μg/mL) [25, 26], suggesting potentially biofilm-active local antibiotic exposure. No antibiotic-related adverse events requiring treatment modification occurred during the study period. None of the patients experienced elevated serum urea or creatinine levels attributable to antibiotic therapy.

## Discussion

The treatment of acute PJI remains a significant challenge for orthopaedic surgeons. Although DAIR is the primary method for treating early postoperative and late acute hematogenous infections, its efficacy has been highly controversial [12]. As mentioned above, previous studies have indicated that the success rate of DAIR varies widely. A review of DAIR-related literature and acute PJI cases found that the success rate of DAIR was as low as 46% [27]. Given the unstable efficacy of DAIR, there is a need to explore additional strategies that may optimize infection management. In this study, following thorough debridement, VSD was applied to cover the surgical wound, aiming to facilitate wound management and reduce potential catheter-related concerns. Additionally, local antibiotic administration to the joint cavity was incorporated with systemic antibiotic therapy as part of a multimodal treatment strategy, with favorable preliminary infection-control and functional outcomes observed in this case series.

Wound infection is one of the main causes of surgical failure, and accelerating wound healing is critical for preventing infection recurrence. In addition, the accumulation of exudates and necrotic tissues in the joint delays the healing process and impairs joint function recovery. Numerous studies have shown that VSD technology reduces tissue edema, improves local microcirculation, and promotes the healing process [28–30]. Enhancing local blood supply helps in toxin clearance and facilitates granulation tissue formation by increasing the gradient of oxygen and nutrients [31, 32]. Furthermore, neovascularization delivers more immune cells and antibiotics, ultimately enhancing bacterial clearance and promoting tissue repair. Kelm et al. reported the application of VSD in early hip prosthetic infections, which provides supportive evidence for the potential role of wound management strategies in PJI treatment [33]. In the DVIA technique, VSD is applied to seal the wound, preventing exposure, maintaining a protected wound environment, and promoting healing. Additionally, before each intra-articular antibiotic infusion, joint effusion was aspirated, reducing joint swelling and promoting both wound recovery and restoration of joint function.

Biofilm formation on the metal surface of prostheses is a major challenge in the treatment of PJI [14]. Biofilm is a dense structure formed by bacterial communities within an extracellular polymeric matrix, exhibiting high antibiotic resistance [34]. Despite thorough debridement of the prosthesis and surrounding tissues during surgery, the risk of bacterial residue and re-adhesion remains. The anatomical characteristics of the joint limit its blood supply, and both infection and surgery may further impair microcirculation. Therefore, relying solely on systemic antibiotic treatment, which typically results in antibiotic concentrations in the joint cavity significantly lower than those in the serum, fails to achieve MBEC [16]. Thus, intra-articular drug delivery has been proposed as a potential strategy to increase local antibiotic exposure [15]. Existing studies have explored the local application of antibiotics in the form of beads, sponges, and powders; however, their efficacy in PJI has not been fully verified. Although some studies have used antibiotic-loaded polymethyl methacrylate (PMMA) beads or calcium sulfate beads in DAIR [35, 36], demonstrating some inhibitory effects on infection, PMMA, as an indigestible foreign particle, can cause tissue damage, whereas calcium sulfate beads may lead to ectopic ossification and hypercalcemia. Therefore, local drug delivery without the need for novel drug carriers may be an ideal treatment option. Previous studies have shown that direct drug delivery into the joint cavity increases antibiotic concentrations, which are much higher than those achieved through intravenous administration, and can maintain the intra-articular antibiotic levels above the minimal inhibitory concentration (MIC) for up to 24 h, effectively controlling the infection [15, 16]. Moreover, this local drug delivery method maintains lower systemic antibiotic concentrations, thereby reducing hepatic and renal toxicity [37]. Multiple teams have used pathogen-specific antibiotics to lavage the joint post-DAIR, which improved the success rate of early infection treatment [38, 39]. A recent systematic review summarizing 15 studies involving 631 PJI cases treated with various surgical procedures combined with intra-articular antibiotic infusion reported an overall failure rate of approximately 11% (Table 4) [40]. A narrative review focusing on intra-articular infusion for PJI following hip arthroplasty summarized favorable infection-control outcomes when intra-articular antibiotics were used as adjuncts to DAIR or revision arthroplasty, along with low rates of systemic toxicity [41]. Other retrospective studies have reported favorable outcomes, with 88% infection-free survival after single-stage revision combined with intra-articular antibiotic infusion during mid- to long-term follow-up [42]. Consistent with these reports, the intra-articular vancomycin concentrations exceeded previously reported MBEC thresholds in our exploratory pharmacokinetic assessment, suggesting that DVIA may achieve high local antibiotic exposure. Meanwhile, compared with previous reports focusing primarily on intra-articular antibiotic delivery, DVIA integrates VSD-based wound management with intra-articular antibiotic infusion, representing an incremental modification combining local antibiotic exposure and wound management during DAIR. Preliminary clinical outcomes suggest that this multimodal approach is safe and feasible, warranting further prospective studies to optimize protocols and clarify the independent contribution of VSD.

**Table 4:** Characteristics of previous studies involving intra-articular antibiotic infusion or negative-pressure wound therapy for PJI and the present DVIA technique

Study	Infection type	Procedure	Local treatment	Local antibiotic concentration	Functional outcomes	Main contribution	Outcome
Mu et al. [17]	PJI occurring within 3 months after primary total joint arthroplasty	DAIR	Intra-articular antibiotic infusion	Not reported	Not reported	Demonstrated feasibility of local antibiotic delivery after DAIR	Success rate 88.57%
Pruk et al. [43]	Acute hematogenous knee PJI	DAIR	Intra-articular antibiotic infusion	Not reported	Not reported	Explored IA infusion combined with DAIR	Success rate 86.66%
Kelm et al. [33]	Early hip prosthetic infections	Revision surgery with debridement and component exchange when feasible	Vacuum-assisted closure	Not applicable	Not reported	Demonstrated the role of negative-pressure therapy in infection management after surgical debridement	Success rate 92.9%
Bruyninckx et al. [40]	PJI treated with IA antibiotic infusion (systematic review)	Various surgical procedures including DAIR and revision arthroplasty	Adjunctive or standalone intra-articular antibiotic infusion	Not systematically reported	Not consistently reported	Summarized current evidence on IA antibiotic infusion	Overall failure rate approximately 11%
Present study	Acute PJI	DAIR	Intra-articular antibiotic infusion + VSD	Exploratory intra-articular vancomycin concentration assessment	Serial functional outcome assessment	Integrated IA antibiotic delivery with VSD-assisted wound management and provided a detailed infusion protocol and preliminary clinical observations	Infection control rate 90.32%

Prolonged intra-articular catheter retention may raise concerns regarding retrograde infection. In our protocol, drainage tubes were placed under strict sterile conditions and accessed only intermittently for aspiration and antibiotic infusion, limiting environmental exposure. The VSD dressing further protected the tubes from contamination. No catheter-tract infection or clinical/microbiological evidence of retrograde infection was observed in our cohort. CRP and ESR, commonly used to monitor infection, decreased following DVIA treatment. Transient postoperative increases in some patients likely reflected normal surgical inflammation, but levels rapidly declined thereafter, consistent with infection control during follow-up.

Our study has several limitations. First, it is a single-center retrospective case series with a relatively small sample size (*n* = 31) and a short follow-up period (1 year). Extended follow-up studies (2–5 years) with larger cohorts are needed to further validate the durability of the DVIA technique and evaluate long-term outcomes regarding infection recurrence, implant survival, and joint function recovery. Second, serial laboratory monitoring data, including CRP/ESR and antibiotic concentration measurements, were available in a subset of patients because of the retrospective nature of data collection. These longitudinal observations provide supportive evidence of treatment response but warrant further validation in larger prospective cohorts with standardized monitoring protocols. Finally, as a preliminary technical series, DVIA combines VSD with intra-articular antibiotic infusion, and the independent contribution of VSD to clinical outcomes cannot be determined in this study. Further multicenter prospective controlled studies are warranted to validate the technique and clarify the specific role of VSD.

In conclusion, we describe an integrated treatment strategy for acute PJI: DVIA. This technique integrates DAIR procedures with intra-articular antibiotic administration and VSD technology, providing a structured approach for wound management, local antibiotic delivery, and infection control. In this case series, DVIA was associated with favorable preliminary clinical outcomes, including favorable infection control, reductions in inflammatory markers, and restoration of joint function. These findings suggest that DVIA is a feasible adjunctive strategy, warranting further multi-center, prospective studies with larger cohorts and longer follow-up to confirm its clinical utility and optimize protocols.

## Data Availability

The datasets used and/or analyzed during the current study are available from the corresponding author upon reasonable request.
